# Sperm competition and the evolution of sperm design in mammals

**DOI:** 10.1186/1471-2148-11-12

**Published:** 2011-01-13

**Authors:** Maximiliano Tourmente, Montserrat Gomendio, Eduardo RS Roldan

**Affiliations:** 1 Reproductive Ecology and Biology Group, Museo Nacional de Ciencias Naturales (CSIC), José Gutiérrez Abascal 2, 28006 Madrid, Spain; 2 Laboratorio de Biología del Comportamiento, Facultad de Ciencias Exactas, Físicas y Naturales, Universidad Nacional de Córdoba, Vélez Sarsfield 299, X5000JJC Córdoba, Argentina

## Abstract

**Background:**

The influence of sperm competition upon sperm size has been a controversial issue during the last 20 years which remains unresolved for mammals. The hypothesis that, when ejaculates compete with rival males, an increase in sperm size would make sperm more competitive because it would increase sperm swimming speed, has generated contradictory results from both theoretical and empirical studies. In addition, the debate has extended to which sperm components should increase in size: the midpiece to accommodate more mitochondria and produce more energy to fuel motility, or the principal piece to generate greater propulsion forces.

**Results:**

In this study we examined the influence of sperm competition upon sperm design in mammals using a much larger data set (226 species) than in previous analyses, and we corrected for phylogenetic effects by using a more complete and resolved phylogeny, and more robust phylogenetic control methods. Our results show that, as sperm competition increases, all sperm components increase in an integrated manner and sperm heads become more elongated. The increase in sperm length was found to be associated with enhanced swimming velocity, an adaptive trait under sperm competition.

**Conclusions:**

We conclude that sperm competition has played an important role in the evolution of sperm design in mammals, and discuss why previous studies have failed to detect it.

## Background

Sperm competition occurs when females mate with more than one male in each sexual cycle and sperm from rival males compete to fertilize the ova [1]. A large body of evidence has accumulated over the last few years showing that sperm competition is a potent evolutionary force that has shaped many reproductive traits [2-4]. An almost universal response to sperm competition across taxa is an increase in sperm numbers, which enhances males' fertilization success in competitive contexts [5-8].

Early theoretical models proposed that the increase in sperm numbers under sperm competition was achieved at the expense of a reduction in their size, leading to the evolution of tiny sperm [9,10]. The presumed trade-off between sperm numbers and size is one of the most widely accepted assumptions in studies of gamete evolution, particularly in relation to the evolution of anisogamy [9,11]. Most empirical studies have therefore assumed that a positive relationship between sperm numbers and size is evidence against the predicted trade-off, and following this reasoning it has been concluded that there is no support for the sperm size/number trade-off except in cases of sperm gigantism [12,13] (reviewed in [14]). However, sperm competition game models are not based on the "direct trade-off", which assumes a fixed budget for ejaculate expenditure and predicts a trade-off between sperm size and numbers [15]. Instead, such models are based on the "indirect trade-off" which assumes a fixed budget for male reproductive activity and proposes that investment in ejaculates is traded-off against energy spent on mate acquisition. Irrespective of this distinction, it is assumed that ejaculate expenditure is the product of the number of sperm and their size [14]. Models developed by Parker [16] considered different advantages that could derive from an increase in sperm size, and concluded that, if the main selective benefit is that sperm become more competitive, sperm competition should not select for an increase in sperm size. Recent models [15] suggest that, even under the indirect trade-off, sperm size and number do effectively trade-off directly, even though both can increase with levels of sperm competition as the total investment on the ejaculate increases. Other types of theoretical models used physical and biomechanical principles and concluded that, in the micro-environment in which sperm perform, a relationship between sperm size and competitive ability (swimming velocity) is unlikely [17]. These theoretical models had a profound influence in this field.

Contrary to these theoretical predictions, an alternative hypothesis proposed that, if an increase in size conferred a competitive advantage to sperm, sperm competition should select simultaneously for an increase in sperm numbers and size, and no trade-off between these two traits should be expected [18]. Preliminary analyses with the evidence available at the time suggested that sperm competition did favour an increase in sperm size which resulted in faster swimming speeds [18].

This hypothesis generated an intense controversy which stimulated a great deal of work on the topic. Empirical work produced an increasing amount of evidence showing that levels of sperm competition were associated with increases in sperm size when interspecific studies were carried out on taxa as diverse as birds, frogs, fish, butterflies, moths, nematodes (reviewed in [19]) and snakes [20]. In some species in which females posses sperm storage organs, this relationship did not become apparent until the size of the female storage organ was taken into account [5,21] (reviewed in [22]).

Different hypotheses were proposed to explain the functional significance of increases in the length of different sperm components: an increase in the flagellum would increase the thrust needed to propel the sperm forward [18], an increase in midpiece volume would increase the amount of energy to fuel sperm motility [20,23,24], and an elongation of the sperm head would reduce the drag experienced by the sperm cell resulting in an increase in sperm swimming velocity [25]. It has also been suggested that the ratios between different sperm components could influence sperm swimming velocity [25] and, in particular, that the ratio sperm head length/flagellum length should play an important role [17].

Recent studies in mammals [19], birds [26], and fish [27] have also provided evidence that sperm size is associated with sperm swimming speed, after phylogenetic effects are taken into account, lending further support to the original hypothesis [18]. Furthermore, intraspecific studies have shown that sperm swimming speed is the main determinant of fertilization success both in competitive [28,29] and non-competitive [30-32] contexts.

However, the evidence linking sperm competition levels and sperm size in mammals remains contentious due to inconsistencies between studies. An analysis using a large sample of species reported a relationship between levels of sperm competition and sperm size, but the relation did not remain significant after phylogenetic effects were taken into account [33]. Because this study included many more species that others, it has been generally assumed that the information provided was more reliable and, as a consequence, it is widely accepted that no relationship exists between sperm competition and sperm length among mammals (see, for example, review in [34]). In addition, further studies on mammals reported either no relationship between sperm competition and sperm size [35], or an effect exclusively on midpiece volume [23,24].

The aim of this study was to carry out the most extensive study to date of the relationship between levels of sperm competition and sperm design in mammals using all the information currently available, which includes a much larger data set and better resolved phylogenies than those used in previous studies. In addition, we examine the relationship between sperm size and sperm swimming velocity, controlling for phylogenetic effects, in a larger data set than previously reported.

## Results

In the study sample of 226 mammals (see additional file 1), total sperm length ranged from 28 μm to 258 μm (CV = 43.8 ± 2.6). The head length accounted for a mean 9.4% of the total sperm length, and it ranged from 3.0 to 15.2 μm (CV = 29.5% ± 0.2). Midpiece length represented a mean 19.9% of the total sperm length (range 3.0-103.1 μm) and it showed higher interspecies variability (CV = 71.2% ± 0.9) than any other sperm component. The principal piece accounted for 70.5% of the total sperm length (range: 15.6-142.6 μm; CV = 41.7% ± 1.9). Body mass and testes mass were extremely variable (CV = 450.53% ± 29928.7 and 494.17% ± 27.6, respectively) when compared to sperm dimensions. On the other hand, relative testes mass, which ranged from 0.07 to 5.23, presented a variability (CV = 84.1% ± 0.1) closer to that seen for sperm dimensions. A summary of information on body mass, relative testes mass and sperm dimensions is shown in additional file 2.

**Figure 1:**
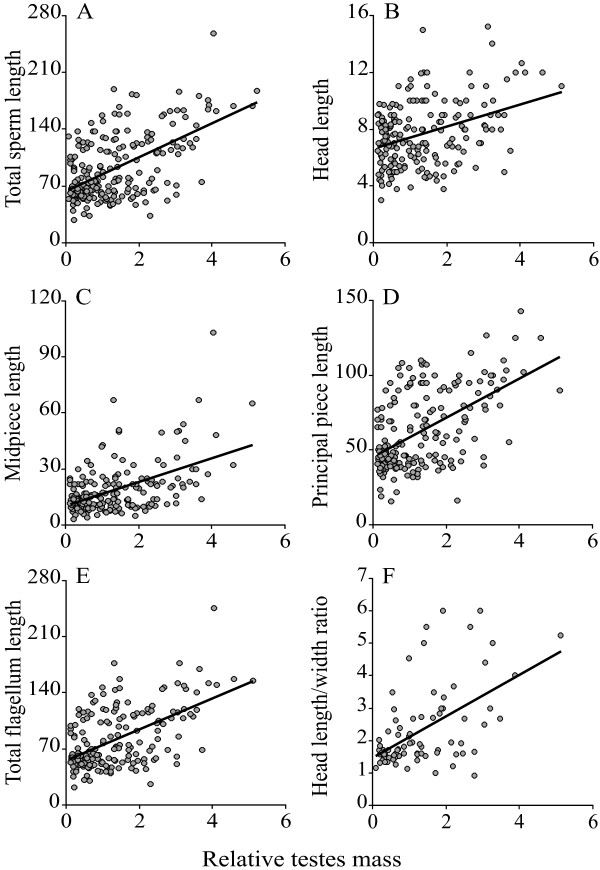
**Relation between sperm competition and sperm dimensions in eutherian mammals**. Relations between relative testes mass and (A) total sperm length (μm), (B) sperm head length (μm), (C) sperm midpiece length (μm), (D) sperm principal piece length (μm), (E) total sperm flagellum length (μm), and (F) sperm head length/width ratio, among eutherian mammals.

We found a significant positive association between testes mass corrected for body mass (thereafter, relative testes mass) and total sperm length after controlling for phylogenetic effects (Table 1, Figure 1A). However, total sperm length was not associated with body mass.

**Table 1:** Relations between sperm competition, sperm dimensions and swimming velocity across Eutherian mammals

Dependent variable	Predictor	Slope	*F*	*p*	*λ*	*r*	CLs	n
total sperm length	body mass	-8.21	1.06	0.3044	0.999 ^*, n.s.^	0.07	-0.06 to 0.20	226
	testes mass	8.24	3.99	**0.0471**		0.13	**0.00 to 0.26**	
head length	body mass	0.74	0.39	0.5317	0.946 ^*, n.s.^	0.05	-0.10 to 0.19	194
	testes mass	0.86	6.53	**0.0114**		0.18	**0.04 to 0.33**	
midpiece length	body mass	-2.63	0.14	0.7116	0.945 ^*, n.s.^	0.03	-0.12 to 0.17	194
	testes mass	3.18	3.41	0.0665		0.13	-0.01 to 0.27	
principal piece length	body mass	-7.57	2.04	0.1547	0.956 ^*, n.s.^	0.10	-0.04 to 0.25	194
	testes mass	7.32	5.62	**0.0188**		0.17	**0.03 to 0.31**	
total flagellum length	body mass	-8.99	0.89	0.3456	0.999 ^*, n.s.^	0.07	-0.07 to 0.21	194
	testes mass	9.61	5.32	**0.0222**		0.16	**0.02 to 0.31**	
head length/head width	body mass	-0.50	0.14	0.7076	0.783 *, *	0.04	-0.18 to 0.27	79
	testes mass	0.56	5.72	**0.0193**		0.26	**0.05 to 0.50**	
straight line velocity	body mass	-39.86	2.08	0.1624	0.880 ^n.s., n.s.^	0.29	-0.11 to 0.71	26
	testes mass	45.71	10.56	**0.0035**		0.56	**0.23 to 1.04**	
straight line velocity	total sperm length	0.72	29.66	**<0.0001**	<0.001 ^n.s., n.s.^	0.74	**0.55 to 1.37**	26
straight line velocity	head length/flagellum length	-635.59	10.55	**0.0047**	<0.001 ^n.s., n.s.^	0.62	**0.23 to 1.21**	19

Phylogenetically controlled multiple regression analyses revealing the effect of relative testes mass on sperm dimensions and sperm velocity, and the effect of sperm length and the proportion between sperm head and flagellum on sperm velocity. The superscripts following the *λ *value indicate significance levels (n.s. *p *> 0.05; * *p *< 0.05) in a likelihood ratio tests against models with *λ *= 0 (first position) and *λ *= 1 (second position). The effect size *r *calculated from the *F *values and its non-central 95% confidence limits (CLs) are presented. Confidence intervals excluding 0 indicate statistically significant relationships. The *p *values and CL that indicate statistical significance are shown in bold. Abbreviations: n: number of species in each analysis.

The same pattern emerged when the different sperm components (head, midpiece, principal plus terminal piece, and flagellum length) were analyzed separately, since all of them became longer as relative testes mass increased, but were unrelated to body mass after controlling for phylogenetic effects (Table 1, Figure 1B-E). The strength of the relationship with relative testes mass was similar for all sperm components except for midpiece length which was just below significance level. In addition, as relative testes mass increased sperm heads became more elongated (measured as the ratio head length/head width) (Table 1, Figure 1F).

**Figure 2:**
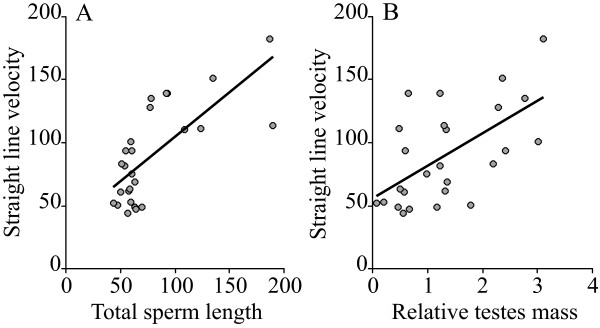
**Relations between sperm velocity, sperm size and sperm competition in eutherian mammals**. Relations between (A) sperm straight-line swimming velocity (μm/s) and total sperm length (μm), and (B) sperm straight line swimming velocity and relative testes mass, in 26 species of eutherian mammals.

Total sperm length showed a strong positive association with sperm swimming velocity (Table 1, Figure 2A). Sperm swimming velocity was also significantly associated with relative testes mass, but not with body mass (Table 1, Figure 2B). It is worth noting that relative testes mass showed a stronger association with sperm swimming velocity than with the size of any sperm component after controlling for phylogenetic effects, and that the relationship between sperm size and sperm swimming velocity is highly significant.

**Figure 3:**
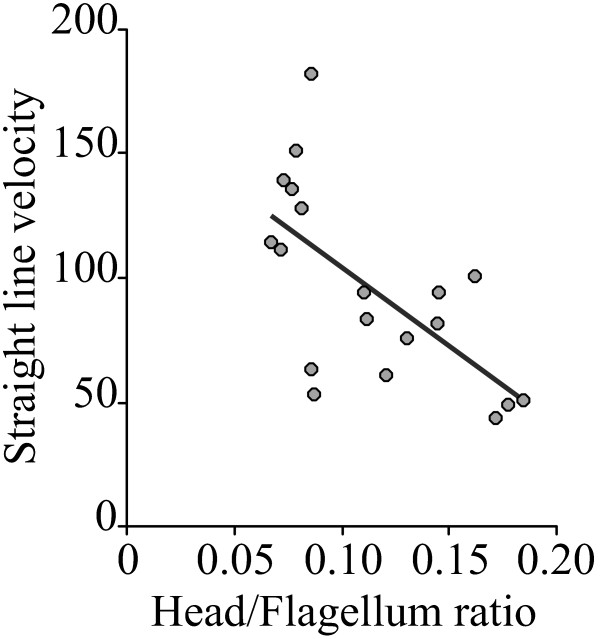
**Relation between sperm velocity and sperm head/flagellum ratio in eutherian mammals**. Relations were examined between sperm straight-line swimming velocity (μm/s) and the proportion between sperm head length (μm) and sperm total flagellum length (μm), in 19 species of eutherian mammals.

The ratio between head length and total flagellum length was significantly associated with straight line velocity (Table 1, Figure 3) so that as the proportion of the flagellum length in relation to head length decreased, sperm velocity also decreased.

## Discussion

In the present study we found evidence suggesting that, in mammals, sperm competition is associated with an increase in total sperm length, which results from an increase in size of all sperm components: head, midpiece, principal plus terminal piece and, hence, flagellum. All relationships between relative testes size (a proxy of sperm competition levels) and sperm dimensions remained significant after controlling for phylogenetic effects, except for the midpiece, which was just below significance level.

The use of relative testes mass as an index of levels of sperm competition is widely accepted, since inter-specific phylogenetic analyses and intra-specific comparisons have shown that both are closely associated (reviewed in [8,36]). In addition, artificial selection experiments have shown that the relationship is causal, since experimental increases in sperm competition levels lead to increases in relative testes size [37]. Recent interspecific analyses, controlling for other variables also believed to influence testes size, show that relative testes mass is clearly related to levels of sperm competition in mammals [38]. The reason why an increase in relative testes size, and therefore sperm production, seems a universal response to levels of sperm competition is that in most taxa sperm numbers influence fertilization success and enhance male fertilization success in competitive contexts [5-8,36].

Our results contrast with those reported by Gage and Freckleton [33] who also found clear relationships between sperm length, and the size of the different sperm components, and relative testes size, although these relationships were lost after controlling for phylogenetic effects. The reasons for this discrepancy may have to do with the fact that our study has a much larger sample size (226 species in our study *versus *83 species in that by Gage and Freckleton [33]) and that these authors used an incorrect grouping of some species (e.g., giraffe was included in Cervidae, *Theropithecus gelada *was placed in the same group with *Erythrocebus patas *and *Cercopithecus aethiops *rather than with *Papio*, and *Homo sapiens *was separated from a group formed by *Gorilla, Pan *and *Pongo*). According to Purvis and Garland [39] these topological errors may affect the performance of phylogenetic control methods, to "an unknown and potentially serious degree".

However, we believe that the main reason for the disparity is that Gage and Freckleton [33] used a very poorly resolved phylogeny that included 12 "soft" polytomies" (i.e., polytomies not due to true simultaneous speciation events but to lack of information about branching pattern) encompassing 57 out of the 83 (69%) species in their study. The reason for this high number of polytomies is that they used a phylogeny which was only resolved up to the family level [40]. In contrast, the phylogeny we used in this study includes only one soft polytomy that affects 3 out of 226 species (1.3%) because we have integrated phylogenetic information for specific groups to avoid the problems associated with low resolution.

There has been much discussion about the effect of polytomies on phylogenetic control methods, and while some authors conclude that a low quality phylogenetic reconstruction is preferable to no phylogenetic control at all [41,42], most studies agree that soft polytomies cause analytical problems for these kind of methods [39,43,44]. Moreover, although these studies recommend possible solutions in the form of analytical methods that reduce degrees of freedom [39,43], estimative approaches using complementary variables [45], random sampling of constrained phylogenies [46], and incorporation of phylogenetic uncertainty into the model via Bayesian methods [47], these approaches have lower statistical power when compared with an analysis that uses a better resolved phylogeny. It is unclear whether Gage and Freckleton [33] used any of these methods to account for the lack of resolution of their phylogeny. The contrast between results (and conclusions) obtained with phylogenies varying in the degree of resolution highlights the need to obtain detailed phylogenetic information to avoid losing meaningful biological relationships when controlling for phylogenetic effects.

To directly examine the possible impact of a poorly resolved phylogeny, we re-analyzed the data in Gage and Freckleton [33] using the detailed phylogenetic reconstruction we used for this study. When doing so, we realized that data for a very few species in Gage and Freckleton's dataset were in fact inadequate. We decided not to include these data to avoid further errors in the analyses. Out of a total of 83 species in the study by Gage and Freckleton [33] we chose to exclude 5 species for the following reasons. Gage and Freckleton [33] included only one species of marsupial (*Antechinus stuartii*) despite the fact that data for many species of marsupials were available at the time of their study [2,48]. The reasons why Gage and Freckleton selected only one species of marsupial is not given in their work and we felt that inclusion of only one species of this clade seriously biased the analysis; the inclusion of a single species from a distantly related clade could affect the outcome of phylogentic analyses. Furthermore, marsupials have a different reproductive biology so the predictions in relation to sperm size are likely to differ. Future studies should address evolution of marsupial sperm taking these factors into consideration. The analysis originally carried out by Gage and Freckleton [33] also included a single cetacean species (*Megaptera novaeangliae*). We decided not to include it in the analyses for the same reasons (i.e., there was information for more cetacean species [48,49] and it was not clear why this particular species was chosen), plus the fact that in cetaceans the scaling of organ/body size is under rather unique constraints (in particular, the testes) due to the selection on streamlined bodies in marine environments. Finally, we also did not include three ungulate species: two species (*Cervus elaphus, Equus grevyi*) were not considered because data on sperm size came from "approximations" calculated from drawings that had no scale (data originally from [48]) whereas one species (*Tayassu tajacu*) was not included because data come from measurements of testis sections (data originally from [48]) and this ignores the post-testicular changes in morphology experimented by spermatozoa.

Thus, when we carried out the re-analysis using the data in the original study by Gage and Freckleton (with the exclusion of questionable data from only 5 out of 83 species) and a well-resolved phylogeny, we found strong positive associations between relative testes size and all sperm components. To verify that the outcome of this re-analysis was not due to the exclusion of the 5 species, we also re-analyzed this dataset using the original phylogeny used by Gage and Freckleton [33]. The results showed no significant association between relative testes size and sperm dimensions (with the exception of head length, which showed a significant association). Thus, when controlling for phylogenetic effects, the same dataset revealed significant relationships with a well-resolved phylogeny which were lost when a poorly-resolved phylogeny was used. We conclude that the lack of association between relative testes size and sperm dimensions in the study by Gage and Freckleton [33] was due mainly to the use of a poorly-resolved phylogeny. Therefore, and contrary to the conclusion by Gage and Freckleton [33], our analyses allow us to conclude that the relationship between sperm dimensions and sperm competition in mammals remains robust after adequately controlling for phylogenetic effects.

There are also some discrepancies between our results and those in other studies on mammals. They can be explained, at least partly, by the different representation of species from different orders in the data sets used. A study on bats found no relationships between levels of sperm competition and sperm dimensions [35]. Because female bats store sperm during hibernation, the need for sperm to survive inside the female reproductive tract for up to several months could imply that in bats sperm competition favours sperm traits which enhance their longevity, rather than their swimming speed. Studies by Dixson and colleagues failed to find relationships between levels of sperm competition and sperm length, or length of various sperm components, first in primates [23] and, subsequently, in mammals [24]. However, since their data derived from samples collected from caudae epididymides during post-mortem dissections of their zoo animals, it consisted primarily of artiodactyls and primates, and included a low number of carnivores and only two species of rodents. Thus, the lack of a relationship between levels of sperm competition and the dimensions of different sperm components found in these studies could be due to an over representation of artiodactyls, carnivores and primates which have lower diversity in sperm dimensions [50]. Intriguingly, Dixson and colleagues found a relationship between sperm competition levels and sperm midpiece volume [23,24]. The reason why midpiece volume but not midpiece length showed a significant relation is not clear, since data show that there is very little variation in midpiece length in artiodactyls, carnivores and primates (see additional file 1). It may be that the width of the midpiece accounts for most of the variation in midpiece volume because of the way it was calculated in that study.

Sperm competition seems to act on all sperm components in an integrative manner, because they are all functionally important and may all contribute in complementary ways to enhance sperm swimming velocity: an increase in midpiece size probably increases energy output [23], and longer principal pieces will generate greater thrust to propel the sperm along the female reproductive tract [18] and generate additional energy [19]. In addition, our study also found that sperm competition levels are also associated to the degree of elongation of the sperm head, a trait which has received little attention despite the major role it could play in sperm hydrodynamics. Recent intraspecific studies have shown that the degree of elongation of the sperm head has the strongest impact upon sperm swimming velocity [25], because it reduces the degree of drag and thus the energy needed for sperm movement [51]. Thus, relatively subtle differences in the shape of the sperm head seem to have a great impact upon swimming performance.

We have also been able to show that as sperm become longer, sperm swimming velocity increases. These findings expand on previous work [19] and agree with those recently reported for birds [26], fish [27] and sea urchin [52]. Furthermore, we have been able to reveal a direct relationship between levels of sperm competition and sperm swimming speed, a link that other studies have failed to find [26]. Interestingly, relative testes size is more strongly associated with sperm swimming velocity than it is with the size of any sperm component, and the relationship between sperm size and sperm swimming velocity is by far the strongest of all the relationships found. This means that relatively small changes in the size of all sperm components and the shape of the head have a major impact on sperm swimming velocity.

Finally, we show that sperm velocity is inversely related to the ratio between head length and flagellum length. Humphries et al. [17] suggested that, since sperm cells are extremely small, thus operating in a low Reynolds number environment, sperm velocity should be determined by the balance between the propulsive force (which would increase with flagellum length) and drag (which would be related to head length). Our findings represent the first comparative evidence supporting this hypothesis, for which a recent intraspecific study also found support [53].

While the picture emerging from interspecific studies seems to support the hypothesis that sperm competition favours increases in sperm size, which enhance sperm swimming velocity resulting in improved fertilization success, the intraspecific studies seem contradictory. Thus, a number of studies show a relationship between sperm dimensions and velocity [25,53,54], whereas a recent review revealed that some intraspecific studies did not find a relationship [17]. This may be due to the fact that the methodology used has enough resolution to detect relationships between species because they differ to a large extent in sperm size, but is insufficient to detect them at the intraspecific level given the small magnitude of differences and the large degree of variation within species and within males. A recent intraspecific study [52] has shown that when sperm data on size and swimming velocity come from the same spermatozoa, the relationship is clear. However, when data on size come from a different sperm subsample than data on sperm swimming velocity (as is the case in most studies) such relationships are not found.

Other intraspecific studies have concluded that small sperm are more competitive (e.g. [55,56]. Studies on insect models suffer from the limitation that so far it has not been possible to evaluate sperm swimming velocity in these species. Thus, the potential relationships between sperm size and sperm swimming velocity cannot be evaluated in these taxa until the technical difficulties are solved. It should also be noted that most intraspecific studies do not actually measure "fertilization success" but rather some measure of paternity success. Thus, they cannot exclude other factors that could be playing a role at any stage after fertilization and during embryo development.

Despite these caveats, it is plausible that the benefits of increasing/decreasing sperm size vary between taxa. Since many intraspecific studies have been carried out on insects, one possibility is that because in this particular group sperm remain in storage organs for long periods of time, variables other than sperm size may be more important at determining fertilization success. Thus, sperm longevity may be the major determinant of fertilization success. If so, small sperm may be at an advantage if they are able to survive for longer, as shown in external fertilizers [31], but the information currently available does not allow to test this hypothesis properly.

The relevance of our findings for other taxa is supported by evidence from a recent review showing that the relationship between sperm competition and sperm size is widespread across taxa [19]. Since female mammals have no sperm storage organs, sperm survive for short periods of time in the female reproductive tract and have to overcome relatively long distances to reach the ova. Under these conditions fertilization success is determined mainly by sperm swimming velocity [32], and there are no confounding effects of co-evolution between sperm size and female storage organs. Thus, it is in this system that the race to fertilize the ova is likely to be the most important underlying mechanism explaining fertilization success in competitive contexts. The outcome is that sperm competition selects for both increased sperm numbers and size, so total ejaculate expenditure increases. The increase in ejaculate expenditure is energetically costly [57,58], but whether it requires a trade-off with mating acquisition, or other life-history traits [e.g. [59-61]], remains to be explored.

## Conclusions

Our findings provide further support to the original hypothesis that the main adaptive value of changes in sperm design under sperm competition is that they increase sperm swimming velocity [18]. Since a number of studies have shown that sperm swimming velocity is a main determinant of fertilization success [19,28,29,32,62], this explains why it is targeted by sexual selection so efficiently.

This study presents phylogenetically-robust evidence that sperm competition in mammals favours an increase in the size of all sperm components and an elongation of the head, which result in faster swimming speeds.

## Methods

The study sample includes all eutherian mammals for which information was obtained. In those cases in which different values for the same species were available from different studies, averages were used. We have not included in the analysis a few species for which the only data available were "approximations from Retzius' illustrations" because they lacked a reference scale (see Methods and footnote to Table 1 in [48]). Additionally, we have not included Chiroptera due to their unusual reproductive traits which include sperm storage for long periods of time in the female tract.

### Sperm competition levels, sperm design and sperm swimming velocity

Data on relative testes mass and sperm dimensions were obtained from the literature for 226 species (39 families) of eutherian mammals (see additional file 1 for data and additional file 3 for references). Sperm dimensions included total sperm length (TSL), head length (HL), head width (HW), midpiece length (MPL), principal piece length (PPL), and total flagellum length (TFL). We also calculated the ratio HL/HW.

Data on sperm swimming velocity from fresh, non-capacitated sperm were obtained from the literature for 26 species (15 families) of eutherian mammals (see additional file 4 for data and additional file 3 for references). We used information on average straight-line velocity (VSL: velocity calculated using the straight-line distance between the beginning and end of the sperm track), since it is a commonly used measure of sperm swimming velocity. In any case, VSL significantly correlates with other sperm swimming parameters such as curvilinear velocity (VCL) or average-path velocity (VAP) [19,32].

### Data analyses

To test whether levels of sperm competition were associated with sperm dimensions, we performed multiple regression analyses using HL, MPL, PPL, TFL, TSL, HL/HW ratio and VSL for all species as dependent variables and relative testes mass (a proxy of sperm competition levels) as predictor. To accurately represent relative testes mass as a measure of sperm competition [38], we performed multiple regression analyses including both log_10_-transformed testes mass and body mass as predictors of sperm dimensions. Since predictor variables were related to each other (thus non orthogonal) (additional file 5), multiple regression analysis was performed using a sequential (Type I) sum of squares, in which the predictor variables were added to the model in the following order: body mass, testes mass.

Species data may not be free of phylogenetic association, since they may share character values as a result of a common ancestry rather than independent evolution [63], and thus may not be truly independent [64]. To control for this phylogenetic inertia, we used a generalized least-squares (GLS) approach in a phylogenetic framework [41]. This method estimates a phylogenetic scaling parameter lambda (*λ*) which represents the transformation that makes the data fit the Brownian motion evolutionary model. If λ values are close to 0, the variables are likely to have evolved independently of phylogeny, whereas λ values close to 1 indicate strong phylogenetic association of the variables. As an advantage, GLS allows a variable degree of phylogenetic correction according to each tested model, accounting for different levels of phylogenetic association between different traits. The estimation of *λ *values and GLS analyses were performed using a code written by R. Freckleton for the statistical package R v.2.8.1 (R Foundation for Statistical Computing 2009) and the maximum likelihood value of *λ *was compared against models with *λ *= 1 and *λ *= 0.

A complete phylogeny for all analyzed species was not available. Therefore, a phylogenetic reconstruction was used (additional file 6). Morphological and molecular trees constructed for the Eutheria were used to determine the phylogenetic position of the higher groups (orders and families), and group-specific phylogenies (both morphological and molecular) were used in the case of the groups that accounted for more than two species (references in additional file 3).

All statistical analyses were conducted with R v.2.8.1, and *p*-values were considered statistically significant at *α *< 0.05. We avoided the use of Bonferroni correction since it increases the chances of committing type II errors [65]. Alternatively, we calculated the effect size *r *from *F *values [66-68] obtained from the GLS model; effect sizes ≥ 0.5 were considered large [69]. Finally, we calculated the non-central confidence limits (CLs) for *r*, which indicate statistical significance if 0 is not contained within the interval [70].

To be able to show values for relative testes mass in the figures, values were calculated by dividing the actual testes mass by the predicted testes mass, which was obtained using the allometric relation between testes mass and body mass predicted by Kenagy and Trombulak [49] for all mammalian species: testes mass = 0.035 × body mass^0.72^. However, because this measure has been criticized as an inaccurate index of sperm competition levels due to allometric problems [71], we have not used it in any of the statistical analyses.

## Authors' contributions

All authors designed and performed research, carried out data compilation and analyses, and participated in preparation of the manuscript. All authors read and approved the final manuscript.

## Authors' information

M. Tourmente enjoyed a Boehringer Ingelheim Travel Grant and a postdoctoral fellowship of the Consejo Nacional de Investigaciones Científicas y Técnicas (CONICET), Argentina. M. Tourmente is currently a postdoctoral researcher funded by the Programa Nacional de Movilidad de Recursos Humanos de Investigación of the Spanish Ministry of Education.

## Supplementary Material

Additional file 1 **Sperm dimensions, body mass, testes mass and relative testes size in 226 species of eutherian mammals**. Abbreviations: HW: sperm head width (μm). HL: sperm head length (μm). MPL: sperm midpiece length (μm). PPL: sperm principal piece length (μm). TFL: total sperm flagellum length (μm). TSL: total sperm length (μm). B mass: body mass (g). T mass: testes mass (g). RTS: relative testes mass. SD: sperm dimensions. Click here for file

Additional file 2 **Mean values and range of corporal and sperm dimensions in 226 species of eutherian mammals**. CV is the coefficient of variation. % TSL is the mean percentage of the total sperm length represented by each sperm component. The mean percentage of increment indicates the difference between the lowest to the highest value among species. Click here for file

Additional file 3 **References for the additional files**. Click here for file

Additional file 4 **Sperm velocity and sperm length in 26 species of eutherian mammals**. Abbreviations: VSL: sperm straight line velocity (μm/s). TSL: total sperm length (μm). Click here for file

Additional file 5 **Relations between testes mass (g) and body mass (g) in 226 species of eutherian mammals**. Values have been converted to Log10 (*p *< 0.0001, *R*^*2 *^= 0.85). The line represented corresponds to the equation of the relation between these two variables published by Kenagy & Trombulak (1986): Log testes mass = (0.72 * Log_10 _body mass) - 1.4559. Click here for file

Additional file 6 **Phylogenetic reconstruction for the 226 eutherian mammal species utilized in the GLS analysis**. Click here for file
