# Supplementary material for: Sperm competition and the evolution of sperm design in mammals
Source: BMC Evol Biol. 2011 Jan 13;11:12. doi: 10.1186/1471-2148-11-12 (PMC3030547; doi:10.1186/1471-2148-11-12)
Supplement: Additional file 3 — References for the additional files. [file 1471-2148-11-12-S3.PDF]

REFERENCES

Adkins, R. M., Walton, A. H., and Honeycutt, R. L. 2003. Higher-level systematics of rodents and divergence time estimates based on two congruent nuclear genes. *Molecular Phylogenetics and Evolution* **26** 409-420.

Agnarsson, I. and May-Collado, L. J. 2008. The phylogeny of Cetartiodactyla: The importance of dense taxon sampling, missing data, and the remarkable promise of cytochrome b to provide reliable species-level phylogenies. *Molecular Phylogenetics and Evolution* **48** 964-985.

Almeida, F. C., Bonvicino, C. R., and Cordeiro-Estrela, P. 2007. Phylogeny and temporal diversiWcation of *Calomys* (Rodentia, Sigmodontinae): Implications for the biogeography of an endemic genus of the open/dry biomes of South America. *Molecular Phylogenetics and Evolution* **42** 449-466.

Almeida, F. F., Leal, M. C., and França, L. R. 2006. Testis morphometry, duration of spermatogenesis, and spermatogenic efficiency in the wild boar (*Sus scrofa scrofa*). *Biol Reprod* **75** 792-799.

Ambriz García, D., Contreras Montiel, J. L., Hernández Pérez, O., Mercado Pichardo, E., Cervantes Reza, F. A., and Rosado García, A. 2003. Estudio comparativp de los testículos, epidídimos, glándulas sexuales accesorias y espermatozoides en tres especies de lagomorfos (*Romerolagus diazi*, *Lepus californicus* y *Oryctolagus cuniculus*). *Acta Zoologica Mexicana* **88** 257-263.

Anderson, J., Nyholt, J., and Dixson, A. F. 2004. Sperm competition affects the structure of the mammalian vas deferens. *Journal of Zoology* **264** 97-103.

Anderson, M. J., Nyholt, J., and Dixson, A. F. 2005. Sperm competition and the evolution of sperm midpiece volume in mammals. *Journal of Zoology* **267** 135-145.

Aurich, C., Seeber, P., and Müller-Schlösser, F. 2007. Comparison of different extenders with defined protein composition for storage of stallion spermatozoa at 5°C. *Reproduction in Domestic Animals* **42** 445-448.

Baena, A., Mootnick, A. R., Falvo, J. V., Tsytskova, A. V., Ligeiro, F., Diop, O. M., Brieva, C., Gagneux, P., O'Brien, S. J., Ryder, O. A., and Goldfeld, A. E. 2007. Primate TNF Promoters Reveal Markers of Phylogeny and Evolution of Innate Immunity. *PLoS One* **2** e621.

Barker, J. M. and Boonstra, R. 2005. Preparing for winter: Divergence in the summer–autumn hematological profiles from representative species of the squirrel family. *Comparative Biochemistry and Physiology A* **142** 32-42.

Batalha, L. M. and Oba, E. 2006. Morphometrical and morphological characterization of capybara (*Hydrochoerus hydrochaeris*) spermatic cells. *Archives of Veterinary Science* **11** 66-72.

Baumber, J. and Meyers, S. A. 2006. Hyperactivated motility in Rhesus macaque (*Macaca mulatta*) spermatozoa. *Journal of Andrology* **27** 459-468.

Beck, R. M., Bininda-Emonds, O. R., Cardillo, M., Liu, F. R., and Purvis, A. 2006. A higher-level MRP supertree of placental mammals. *BMC Evolutionary Biology* **6** 93-107.

Bininda-Emonds, O. R., Cardillo, M., Jones, K. E., MacPhee, R. D., Beck, R. M., Grenyer, R., Price, S. A., Vos, R. A., Gittleman, J. L., and Purvis, A. 2007. The delayed rise of present-day mammals. *Nature* **446** 507-512.

Blottner, S., Schön, J., and Jewgenow, K. 2006. Seasonally activated spermatogenesis is correlated with increased testicular production of testosterone and epidermal growth factor in mink (*Mustela vison*). *Theriogenology* **66** 1593-1598.

Böhm, M. and Mayhew, P. J. 2005. Historical biogeography and the evolution of the latitudinal gradient of species richness in the Papionini (Primata: Cercopithecidae). *Biological Journal of the Linnean Society* **85** 235-246.

Borges, B. N., Paiva, T. S., and Harada, M. L. 2008. Evolution of the SEC1 gene in New World monkey lineages (Primates, Platyrrhini). *Genetics and Molecular Research* **7** 663-678.

Bradley, R. D., Durish, N. D., Rogers, D. S., Miller, J. R., Engstrom, M. D., and Kilpatrick, C. W. 2007. Toward a molecular phylogeny for *Peromyscus*: evidence from mitochondrial cytochrome-b sequences. *Journal of Mammalogy* **88** 1146-1159.

Breed, W. G. 1995. Spermatozoa of murid rodents from Africa: morphological diversity and evolutionary trends. *Journal of Zoology* **237** 625-651.

Breed, W. G. and Taylor, J. 2000. Body mass, testes mass, and sperm size in Murine rodents. *Journal of Mammalogy* **81** 758-768.

Breed, W. G. and Yong, H. S. 1986. Sperm morphology of murid rodents from Malaysia and its possible phylogenetic significance. *American Museum Novitates* **2856** 1-12.

Breed, W. G. 2004. The spermatozoon of eurasian murine rodents: its morphological diversity and evolution. *Journal of Morphology* **261** 52-69.

Cassinello, J., Abaigar, T., Gomendio, M., and Roldan, E. R. S. 1998. Characteristics of the semen of three endangered species of gazelles (*Gazella dama mhorr, G. dorcas neglecta* and *G.cuvieri*). *Journal of Reproduction and Fertility* **113** 35-45.

Castellini, C., Lattaioli, P., Bernardini, M., and Dal Bosco, A. 2000. Effect of dietary a-Tocopheryl Acetate and Ascorbic Acid on rabbit semen stored at 5° C. *Theriogenology* **54** 523-533.

Castellini, C., Pizzi, F., Theau-Clément, M., and Lattaioli, P. 2006. Effect of different number of frozen spermatozoa inseminated on the reproductive performance of rabbit does. *Theriogenology* **66** 2182-2187.

Conroy, C. J. and Cook, J. A. 2000. Molecular systematics of a holartic rodent (*Microtus*: Muridae). *Journal of Mammalogy* **81** 344-359.

Cooper, T. G., Weydert, S., Ching-Hei, Y., Künzl, C., and Sachser, N. 2000. Maturation of epididymal spermatozoa in the nondomesticated Guinea pigs *Cavia aperea* and *Galea musteloides*. *Journal of Andrology* **21** 154-163.

Cox, J. F., Alfaro, V., Montenegro, V., and Rodriguez-Martinez, H. 2006. Computer-assisted analysis of sperm motion in goats and its relationship with sperm migration in cervical mucus. *Theriogenology* **66** 860-867.

Crosier, A. E., Marker, L., Howard, J., Pukazhenthi, B. S., Henghali, J. N., and Wildt, D. E. 2007. Ejaculate traits in the Namibian cheetah (*Acinonyx jubatus*): influence of age, season and captivity. *Reprod. Fertil. Dev.* **19** 370-382.

Cummins, J. M. and Woodall, P. F. 1985. On mammalian sperm dimensions. *Journal of Reproduction and Fertility* **75** 153-175.

Dixson, A. F. and Anderson, M. J. 2004. Sexual behavior, reproductive physiology and sperm competition in male mammals. *Physiology & Behavior* **83** 361-371.

Dubey, S., Salamin, N., Ohdachi, S. D., Barriere, P., and Vogel, P. 2007. Molecular phylogenetics of shrews (Mammalia: Soricidae) reveal timing of transcontinental colonizations. *Molecular Phylogenetics and Evolution* **44** 126-137.

Dukelow, W. R. 1971 Semen and artificial insemination. In: *Comparative Reproduction of Nonhuman Primates.* (ed. Hafez, E. S.), pp 115-117. Springfield, US: Thomas.

Flynn, J. J., Finarelli, J. A., Zehr, S., Hsu, J., and Nedbal, M. A. 2005. Molecular Phylogeny of the Carnivora (Mammalia): Assessing the Impact of Increased Sampling on Resolving Enigmatic Relationships. *Systematic Biology* **54** 317-337.

Ford, F. 2006. A splitting headache: relationships and generic boundaries among Australian murids. *Biological Journal of the Linnean Society* **89** 117-138.

França, L. R. and Godinho, C. L. 2003. Testis morphometry, seminiferous epithelium cycle length, and daily sperm production in Domestic Cats (*Felis catus*). *Biol Reprod* **68** 1554-1561.

Fulton, T. L. and Strobeck, C. 2007. Novel phylogeny of the raccoon family (Procyonidae: Carnivora) based on nuclear and mitochondrial DNA evidence. *Molecular Phylogenetics and Evolution* **43** 1171-1177.

Gage, M. J. G. 1998. Mammalian sperm morphometry. *Proceedings of the Royal Society B: Biological Sciences* **265** 97-103.

Gage, M. J. G. and Freckleton, R. 2003. Relative testis size and sperm morphometry across mammals: no evidence for an association between sperm competition and sperm length. *Proceedings of the Royal Society B: Biological Sciences* **270** 625-632.

Gallardo, M. H., Mondaca, F. C., Ojeda, R. A., Köhler, N., and Garrido, O. 2002. Morphological diversity in the sperms of Caviomnorph rodents. *Journal of Neotropical Mammals* **9** 159-170.

González Moreno, M. C., Cetica, P. D., and Merani, M. S. 2000. *Journal of Primatology* **30** 309-314.

Grzmil, P., Golas, A., Müller, C., and Styrna, J. 2007. The influence of the deletion on the long arm of the Y chromosome on sperm motility in mice. *Theriogenology* **67** 760-766.

Helbig, L., Woodbury, M. R., Haig, J. C., Collins, J., and Barth, A. D. 2007. The seasonal fertility of North American bison (*Bison bison*) bulls. *Animal Reproduction Science* **97** 265-277.

Hoffman, K. 1979. Photoperiod, pineal, melatonin and reproduction in hamsters. *Progress in Brain Research* **52** 397-415.

Holt, W. V., O'Brien, J. K., and Abaigar, T. 2007. Applications and interpretation of computer-assisted sperm analyses and sperm sorting methods in assisted breeding and comparative research. *Reprod. Fertil. Dev.* **19** 709-718.

Howard, J. G., Zhang, Z., Li, D., Huang, Y., Li, G., Zhang, M., Ye, Z., Zhang, J. P., Huang, S., Spindler, R. E., Zhang, H., & Wildt, D. E. 2006 Male reproductive biology in giant pandas in breeding programmes in China. In: *Giant pandas: biology, veterinary medicine and management.* (ed. Wildt, D.; Zhang, A.; Zhang, H.; Janssen, D.; & Ellis, S.), pp 159-197. Cambridge, UK: Cambridge University Press.

Hung, P. H., Baumber, J., Meyers, S. A., and VandeVoort, C. A. 2007. Effects of environmental tobacco smoke in vitro on rhesus monkey sperm function. *Reproductive Toxicology* **23** 499-506.

Immler, S., Moore, H. D., Breed, W. G., and Birkhead, T. R. 2007. By hook or by crook? Morphometry, competition and cooperation in rodent sperm. *PLoS One* **2** e170.

Johnson, S. E., Gordon, A. D., Stumpf, R. M., Overdoff, D. J., and Wright, P. C. 2005. Morphological variation in populations of *Eulemur albocollaris* and *E. fulvus rufus*. *International Journal of Primatology* **26** 1399-1416.

Johnson, W. E., Eizirik, E., Pecon-Slattery, J., Murphy, W. J., Antunes, A., Teeling, E., and O'Brien, S. J. 2006. The Late Miocene Radiation of Modern Felidae: A Genetic Assessment. *Science* **311** 73-77.

Johnston, S. D., Ward, D., Lemon, J., Gunn, I., MacCallum, C. A., Keeley, T., and Blyde, D. 2007. Studies of male reproduction in captive African wild dogs (*Lycaon pictus*). *Animal Reproduction Science* **100** 338-355.

Kenagy, G. J. and Trombulak, C. 1986. Size and function of mammalian tstes in relation to body size. *Journal of Mammalogy* **67** 1-22.

Kjer, K. M. and Honeycutt, R. L. 2007. Site specific rates of mitochondrial genomes and the phylogeny of eutheria. *BMC Evolutionary Biology* **7** 8-17.

Kozdrowski, R. 2007. Assessment of the quality of European hare (*Lepus europaeus*, Pallas 1778) semen with conventional computer methods. *Bulletin of the Veterinary Institute of Pulawy* **51** 569-573.

Kozdrowski, R., Dubiel, A., and Dzimira, S. 2006. Morphology of European brown hare (*Lepus europaeus* Pallas, 1778) semen. *Bulletin of the Veterinary Institute of Pulawy* **50** 383-385.

Lagerkvist, G., Johansson, K., and Lundeheim, N. 1994. Selection for litter size, body weight, and pelt quality in mink (*Mustela vison*): correlated responses. *Journal of Animal Science* **72** 1126-1137.

Lavara, R., Mocé, E., Lavara, F., Viudes de Castro, M. P., and Vicente, J. S. 2005. Do parameters of seminal quality correlate with the results of on-farm inseminations in rabbits? *Theriogenology* **64** 1130-1141.

Lecompte, E., Aplin, K. P., Denys, C., Catzeflis, F., Chades, M., and Chevret, P. 2008. Phylogeny and biogeography of African Murinae based on mitochondrial and nuclear gene sequences, with a new tribal classification of the subfamily. *BMC Evolutionary Biology* **8** 199-220.

Lima, G. L., Barros, F. F., Costa, L. L., Castelo, T. S., Fontenele-Neto, J. D., and Silva, A. R. 2008. Determination of semen characteristics and sperm cell ultrastructure of captive coatis (*Nasua nasua*) collected by electroejaculation. *Animal Reproduction Science* In press.

Lincoln, G. A. 1989. Seasonal cycles in testicular activity in Mouflon, Soay sheep and domesticated breeds of sheep: breeding seasons modified by domestication. *Zoological Journal of the Linnean Society* **95** 137-147.

Liu, X., Wei, F., Li, M., Jiang, X., Feng, Z., and Hu, J. 2004. Molecular phylogeny and taxonomy of wood mice (genus *Apodemus* Kaup, 1829) based on complete mtDNA cytochrome b sequences, with emphasis on Chinese species. *Molecular Phylogenetics and Evolution* **33** 1-15.

Lötter, T. K. and Pillay, N. 2008. Reproduction and postnatal development of the bushveld gerbil *Gerbilliscus* (formerly *Tatera*) *leucogaster* . *Mammalian Biology* **73** 430-437.

Lynch, C. D. 1980. Ecology of the suricate, *Suricata suricatta*, and the yellow mongoose, *Cynictis penilcillata*, with special reference to their reproduction. *Memoirs van die Nasionale Museum, Bloemfontein* **14** 1-145.

Mahony, M. C., Lanzendorf, S., Gordon, K., and Hodgen, G. D. 1996. Effects of Caffeine and dbcAMP on pona pellucida penetration by epididymal spermatozoa of cynomolgus monkeys (*Macaca fascicularis*). *Molecular Reproduction and Development* **43** 530-535.

Malo, A. F., Gomendio, M., Garde, J., Lang-Lenton, B., Soler, A. J., and Roldan, E. R. S. 2006. Sperm design and sperm function. *Biology Letters* **2** 246-249.

Marmi, J., López-Giráldez, J. F., and Domingo-Roura, X. 2004. Phylogeny, evolutionary history and taxonomy of the Mustelidae based on sequences of the cytochrome b gene and a complex repetitive flanking region. *Zoologica Scripta* **33** 481-499.

Martin, D. E., Gould, K. G., and Warner, H. 1975. Comparative morphology of primate spermatozoa using scanning electron microscopy. I. Families Hominidae, Pongidae, Cercopithecidae and Cebidae. *Journal of Human Evolution* **4** 287-292.

Mastroianni, L. and Manson, W. A. 1963. Collection of monkey semen by electroejaculation. *Proceedings of the Society for Experimental Biology and Medicine* **112** 1025-1027.

Matsushima, S., Sakai, Y., and Hira, Y. 1990. Effect of photoperiod on pineal gland volume and pinealocyte size in the Chinese Hamster, *Cricetulus griseus*. *American Journal of Anatomy* **187** 32-38.

Meisner, A. D., Klaus, A. V., and O'Leary, M. A. 2005. Sperm head morphology in 36 species of artiodactylans, perissodactylans, and cetaceans (Mammalia). *Journal of Morphology* **263** 179-202.

Mekasha, Y., Tegegne, A., Abera, A., and Rodriguez-Martinez, H. 2008. Body size and testicular traits of tropically–adapted bucks raised under extensive husbandry in Ethiopia. *Reproduction in Domestic Animals* **43** 196-206.

Menzies, J. I. 1996. A systematic revision of *Melomys* (Rodentia: Muridae) of New Guinea. *Australian Journal of Zoology* **44** 367-426.

Michaux, J., Chevret, P., and Renaud, S. 2007. Morphological diversity of Old World rats and mice (Rodentia, Muridae) mandible in relation with phylogeny and adaptation. *Journal of Zoological Systematics & Evolutionary Research* **45** 263-279.

Minter, L. J. and DeLiberto, T. J. 2008. Seasonal variation in serum testosterone, testicular volume, and semen characteristics in the coyote (*Canis latrans*). *Theriogenology* **69** 946-952.

Miró, J., Lobo, V., Quintero-Moreno, A., Medrano, A., Peña, A., and Rigau, T. 2005. Sperm motility patterns and metabolism in Catalonian donkey semen. *Theriogenology* **63** 1706-1716.

Mollineau, W., Adogwa, A., and García, A. J. 2008. Spermatozoal morphologies and fructose and citric acid concentrations in agouti (*Dasyprocta leprorina*) semen. *Animal Reproduction Science* **105** 378-383.

Mollineau, W., Adogwa, A., Jasper, N., Young, K., and García, G. 2006. The gross anatomy of the male reproductive system of a Neotropical rodent: the Agouti (*Dasyprota leporina*). *Anatomy, Histology and Embriology* **35** 47-52.

Morais, R. N., Mucciolo, R. G., Gomes, M. L., Lacerda, O., Moraes, W., Moreira, N., Graham, L. H., Swanson, W. F., and Brown, J. L. 2002. Seasonal analysis of semen characteristics, serum testosterone and fecal androgens in the ocelot (*Leopardus pardalis*), margay (*L. wiedii*) and tigrina (*L. tigrinus*). *Theriogenology* **57** 2027-2041.

Morgan, C. C. 2008. Geometric morphometrics of the scapula of South American caviomorph rodents (Rodentia: Hystricognathi): Form, function and phylogeny. *Mammalian Biology* In press.

Morrell, J. M. 1997. CASA as an aid to selecting sperm suspensions for artifcial insemination in Callithrix jacchus. *International Journal of Andrology* **20** 287-296.

Mortimer, S. T. and Mortimer, D. 1990. Kinematics of human spermatozoa incubated under capacitating conditions. *Journal of Andrology* **11** 195-203.

Neal, B. R. 1995. The ecology and reproduction of the Short-snouted Elephant-Shrew, *Elephantulus brachyrhynchus*, in Zimbawe with a review of the reproductive ecology of the genus *Elephantulus*. *Mammal Reviews* **25** 51-60.

Nishihara, H., Hasegawa, M., and Okada, N. 2006. Pegasoferae, an unexpected mammalian clade revealed by tracking ancient retroposon insertions. *Proceedings of the National Academy of Sciences* **103** 9929-9934.

O'Leary, M. A. and Gatesy, J. 2008. Impact of increased character sampling on the phylogeny of Cetartiodactyla (Mammalia): combined analysis including fossils. *Cladistics* **24** 397-442.

Oakenfull, E. A., Lim, H. N., and Ryder, O. A. 2000. A survey of equid mitochondrial DNA: Implications for the evolution, genetic diversity and conservation of Equus. *Conservation Genetics* **1** 341-355.

Odihambo, R. O., Makundi, R. H., Leirs, H., and Verhagen, R. 2008. Demography, reproductive biology and diet of the bushveld gerbil *Tatera leucogaster* (Rodentia: Gerbillinae) in the Lake Rukwa valley, south-western Tanzania. *Integrative Zoology* **3** 31-37.

Pant, H. C., Sharma, R. K., Patel, S. H., Shukla, H. R., Mittal, A. K., Kasiraj, R., Misra, A. K., and Prabhakar, J. H. 2003. Testicular development and its relationship to semen production in Murrah buffalo bulls. *Theriogenology* **60** 27-34.

Parapanov, R. N., Nusslé, S., Crausaz, M., Senn, A., Hausser, J., and Vogel, P. 2008. Testis size, sperm characteristics and testosterone concentrations in four species of shrews (Mammalia, Soricidae). *Animal Reproduction Science* In press.

Patil, S. B., Jayaprakash, D., and Shivaji, S. 1998. Cryopreservation of semen of tigers and lions: computerized analysis of the motility parameters of the spermatozoa. *Current Science* **75** 930-935.

Pérez Garnelo, S. S., Delclaux, M., Talavera, C., López, M., and de la Fuente, J. 2003. Use of computerized image analysis in the morphometric characterization of giant panda (*Ailuropoda melanoleuca*) spermatozoa obtained from the epididymis 4 hours postmortem. *Zoo Biology* **22** 355-364.

Pierce, J. D., Ferguson, B. Jr., Salo, A. L., Sawrey, D. K., Shapiro, L. E., Taylor, S. A., and Dewsbury, D. A. 1990. Patterns of sperm allocation across successive ejaculates in four species of voles (*Microtus*). *Journal of Reproduction and Fertility* **88** 141-149.

Pitra, C., Fickel, J., Meijaard, E., and Colin Groves, P. 2004. Evolution and phylogeny of old world deer. *Molecular Phylogenetics and Evolution* **33** 880-895.

Platz, C. C., Wildt, D., Howard, J., and Bush, M. 1983. Electroejaculation and semen analysis and freezing in the giant panda (Ailuropoda melanoleuca). *Journal of Reproduction and Fertility* **67** 9-12.

Poux, C. and Douzery, E. J. 2004. Primate Phylogeny, Evolutionary Rate Variations, and Divergence Times: A Contribution From the Nuclear Gene IRBP. *American Journal of Physical Anthropology* **124** 1-16.

Prothero, D. R. & Foss, S. E. 2007 *The Evolution of Artiodactyls*. Baltimore, USA: JHU Press.

Rijsselaere, T., Maes, D., Hoflack, G., de Kruif, A., and Van Soom, A. 2007. Effect of body weight, age and breeding history on canine sperm quality parameters measured by the Hamilton-Thorne analyser. *Reproduction in Domestic Animals* **42** 143-148.

Robins, J. H., Hingston, M., Mathisoo-Smith, E., and Ross, H. A. 2007. Identifying *Rattus* species using mitochondrial DNA. *Molecular Ecology Notes* **7** 717-729.

Robinson, T. J. and Mathee, C. A. 2005. Phylogeny and evolutionary origins of the Leporidae: a review of cytogenetics, molecular analyses and a supermatrix analysis. *Mammal Reviews* **35** 231-247.

Roldan, E. R. S., Gomendio, M., and Vitullo, A. D. 1992. The evolution of euhterian spermatozoa and underlying selective forces: female selection and sperm competition. *Biological Reviews* **67** 551-593.

Roos, C., Schmitz, J., and Zischler, H. 2004. Primate jumping genes elucidate strepsirrhine phylogeny. *Proceedings of the National Academy of Sciences* **101** 10650-10654.

Rowe, K. C., Reno, M. L., Richmond, D. M., Adkins, R. M., and Steppan, S. J. 2008. Pliocene colonization and adaptive radiations in Australia and New Guinea (Sahul): Multilocus systematics of the old endemic rodents (Muroidea: Murinae). *Molecular Phylogenetics and Evolution* **47** 84-101.

Ruedas, L. A. and Kirsch, J. A. 1997. Systematics of Maxomys Sody, 1936 (Rodentia: Muridae: Murinae): DNALDNA hybridization studies of some Borneo-Javan species and allied Sundaic and Australo-Papuan genera. *Biological Journal of the Linnean Society* **81** 385-408.

Sears, K. E., Finarelli, J. A., Flynn, J. J., and Wyss, A. R. 2008. Estimating body mass in New World monkeys(Platyrrhini, Primates) with a consideration of the Miocene platyrrhine, *Chilecebus carrascoensis*. *American Museum Novitates* **3167** 1-29.

Seddon, J. M. and Baverstock, P. R. 2000. Evolutionary Lineages of RT1.Ba in the Australian *Rattus*. *Molecular Biology and Evolution* **17** 768-772.

Selvaraju, S., Reddy, I. J., Nandi, S., Rao, S. B., and Ravindra, J. P. 2008. Influence of IGF-I on buffalo (*Bubalus bubalis*) spermatozoa motility, membrane integrity, lipid peroxidation and fructose uptake in vitro. *Animal Reproduction Science* In press.

Sharma, A. K. and Gupta, R. C. 1978. Mensuration of spermatozoa from different levels of the reproductive tract of the buffalo-bull (*Bubalus bubalis*). *Annual Biology Animal Biochemistry and Biophysics* **18** 717-720.

Shivaji, S., Peedicayil, J., and Girija Devi, L. 1995. Analysis of the motility parameters of in vitro hyperactivated hamster spermatozoa. *Molecular Reproduction and Development* **42** 233-247.

Sloter, E., Schmitt, T. E., Marchetti, F., Eskenazi, B., Nath, J., and Wyrobek, A. J. 2006. Quantitative effects of male age on sperm motion. *Human Reproduction* **121** 2868-2875.

Springer, M. S. and Murphy, W. J. 2007. Mammalian evolution and biomedicine: new views from phylogeny. *Biological Reviews* **82** 375-392.

Stacheki, J. J., Ginsburg, K. A., and Armant, D. R. 1994. Stimulation of cryopreserved epididymal spermatozoa of the Domestic Cat using the motility stimulants Caffeine, Pentoxifylline, and 2'-Deoxyadenosine. *Journal of Andrology* **15** 157-164.

Stacheki, J. J., Ginsburg, K. A., Leach, R. E., and Armant, D. R. 1993. Computer-assisted semen analysis (CASA) of epididymal sperm from the Domestic Cat. *Journal of Andrology* **14** 60-65.

Steiper, M. E. and Ruvolo, M. 2003. New World monkey phylogeny based on X-linked G6PD DNA sequences. *Molecular Phylogenetics and Evolution* **27** 121-130.

Steklenev, E. P. 1975. Species characteristics of sperm morpholofy of individual members of the Order (Carnivora) in relation to hybridization. *Tsitologiya i Genetika* **9** 142-146.

Steppan, S. J., Adkins, R. M., and Anderson, J. 2004. Phylogeny and divergence-date estimates of rapid radiations in Muroid reodents based on multiple nuclear genes. *Systematic Biology* **53** 533-553.

Steppan, S. J., Adkins, R. M., Spinks, P. Q., and Hale, C. 2005. Multigene phylogeny of the Old World mice, Murinae, reveals distinct geographic lineages and the declining utility of mitochondrial genes compared to nuclear genes. *Molecular Phylogenetics and Evolution* **37** 370-388.

Swann, C. A., Cooper, S. J., and Breed, W. G. 2007. Molecular evolution of the carboxy terminal region of the zona pellucida 3 glycoprotein in murine rodents. *Reproduction* **133** 697-708.

Tibary, A. and Vaughan, J. 2006. Reproductive physiology and infertility in male South American camelids: A review and clinical observations. *Small Ruminant Research* **61** 283-298.

Trainor, B. C., Martin, L. I., Greiwe, K. M., Kuhlman, J. R., and Nelson, R. J. 2006. Social and photoperiod effects on reproduction in five species of *Peromyscus*. *General and Comparative Edocrinology* **148** 252-259.

Trimeche, A., Renard, P., Le Lannou, D., Barrière, P., and Tainturier, D. 1996. Improvement of motility of post-thaw Pottou jackass sperm using Glutamine. *Theriogenology* **45** 1015-1027.

Trimeche, A., Yvon, J. M., Vidament, M., Palmer, E., and Magistrini, M. 1999. Effects of Glutamine, Proline, Histidine and Betaine on post-thaw motility of stallion spermatozoa. *Theriogenology* **52** 181-191.

Van der Horst, G., Seier, J. V., Spinks, A. C., and Hendricks, S. 1999. The maturation of sperm motility in the epididymis and vas deferens of the vervet monkey, *Cercopithecus aethiops*. *International Journal of Andrology* **22** 197-207.

van Staaden, M. J. 1994. *Suricata suricatta*. *Mammalian Species Account* **483** 1-8.

Veron, G., Colyn, M., Dunham, A. E., Taylor, P., and Gaubert, P. 2004. Molecular systematics and origin of sociality in mongooses (Herpestidae, Carnivora). *Molecular Phylogenetics and Evolution* **30** 582-598.

Watts, C. H. and Baverstock, P. R. 1994. Evolution in New Guinean Muridae (Rodentia) assessed by microcomplement fixation of Albumin. *Australian Journal of Zoology* **42** 295-306.

Weisbecker, V. and Schmid, S. 2007. Autopodial skeletal diversity in hystricognath rodents: Functional and phylogenetic aspects. *Mammalian Biology* **72** 27-44.

Weng, Q., Medan, M. S., Xu, M., Tsubota, T., Watanabe, G., and Taya, K. 2006. Seasonal changes in inmunolocalization of Inhibin/Activin subunits and testicular activity in wild male racoon dogs (*Nyctereutes procyionoides*). *Journal of Reproduction and Development* **52** 503-510.

White, D. Jr., Berardinelli, J. G., and Aune, K. E. 2005. Age variation in gross and histological characteristics of the testis and epididymis in grizzly bears. *Ursus* **16** 190-197.

Windberg, L. A., Engeman, R. M., and Bromaghin, J. F. 1991. Body size and condition of coyotes in Southern Texas. *Journal of Wildlife Diseases* **27** 47-52.

Woodall, P. F. and Johnstone, I. P. 1988. Dimensions and allometry of testes, epididymides and spermatozoa in the domestic dog (*Canis familiaris*). *Journal of Reproduction and Fertility* **82** 603-609.

Xing, J., Wang, H., Han, K., Ray, D. A., Huang, C. H., Chemnick, L. G., Stewart, C., Disotell, T. R., Ryder, O. A., and Batzer, M. A. 2005. A mobile element based phylogeny of Old World monkeys. *Molecular Phylogenetics and Evolution* **37** 872-880.

Yoder, A. D. and Yang, Z. 2004. Divergence dates for Malagasy lemurs estimated from multiple gene loci: geological and evolutionary context. *Molecular Ecology* **13** 757-773.

Zenuto, R. R., Vitullo, A. D., and Busch, C. 2003. Sperm characteristics in two populations of the subterranean rodent *Ctenomys talarum* (Rodentia: Octodontidae). *Journal of Mammalogy* **84** 877-885.
