# Supplementary material for: Sperm competition and the evolution of sperm design in mammals
Source: BMC Evol Biol. 2011 Jan 13;11:12. doi: 10.1186/1471-2148-11-12 (PMC3030547; doi:10.1186/1471-2148-11-12)
Supplement: Additional file 5 — Relations between testes mass (g) and body mass (g) in 226 species of eutherian mammals. Values have been converted to Log10 (p < 0.0001, R2 = 0.85). The line represented corresponds to the equation of the relation between these two variables published by Kenagy & Trombulak (1986): Log testes mass = (0.72 * Log10 body mass) - 1.4559. [file 1471-2148-11-12-S5.PDF]

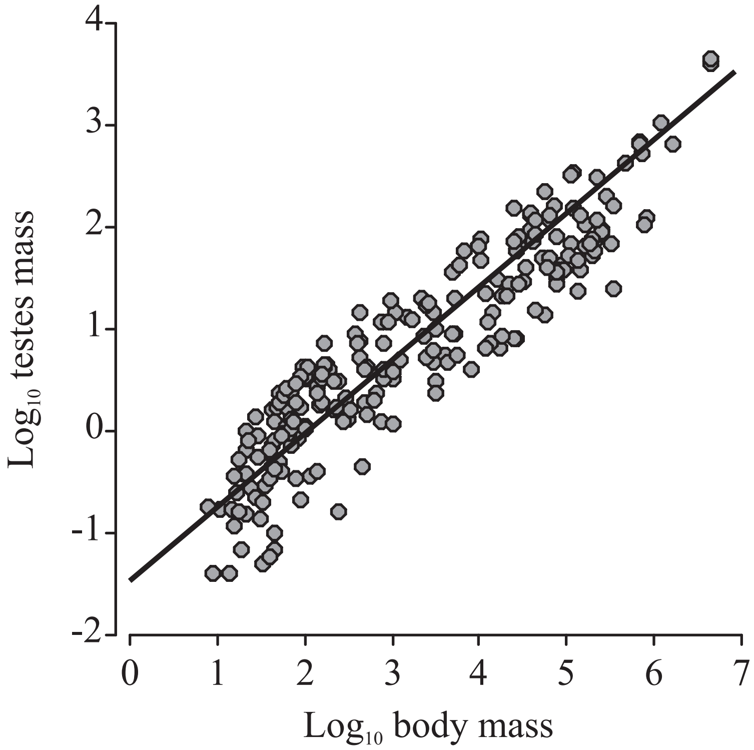

Additional file 4. Relations between testes mass (g) and body mass (g) in 226 species of eutherian mammals. Values have been converted to Log10 (p<0.0001, R^2=0.85). The line represented corresponds to the equation of the relation between these two variables published by Kenagy & Trombulak (1986): Log_{10} testes mass=(0.72 * Log_{10} body mass)-1.4559.
