# Supplementary material for: Sperm competition and the evolution of sperm design in mammals
Source: BMC Evol Biol. 2011 Jan 13;11:12. doi: 10.1186/1471-2148-11-12 (PMC3030547; doi:10.1186/1471-2148-11-12)
Supplement: Additional file 6 — Phylogenetic reconstruction for the 226 eutherian mammal species utilized in the GLS analysis. [file 1471-2148-11-12-S6.PDF]

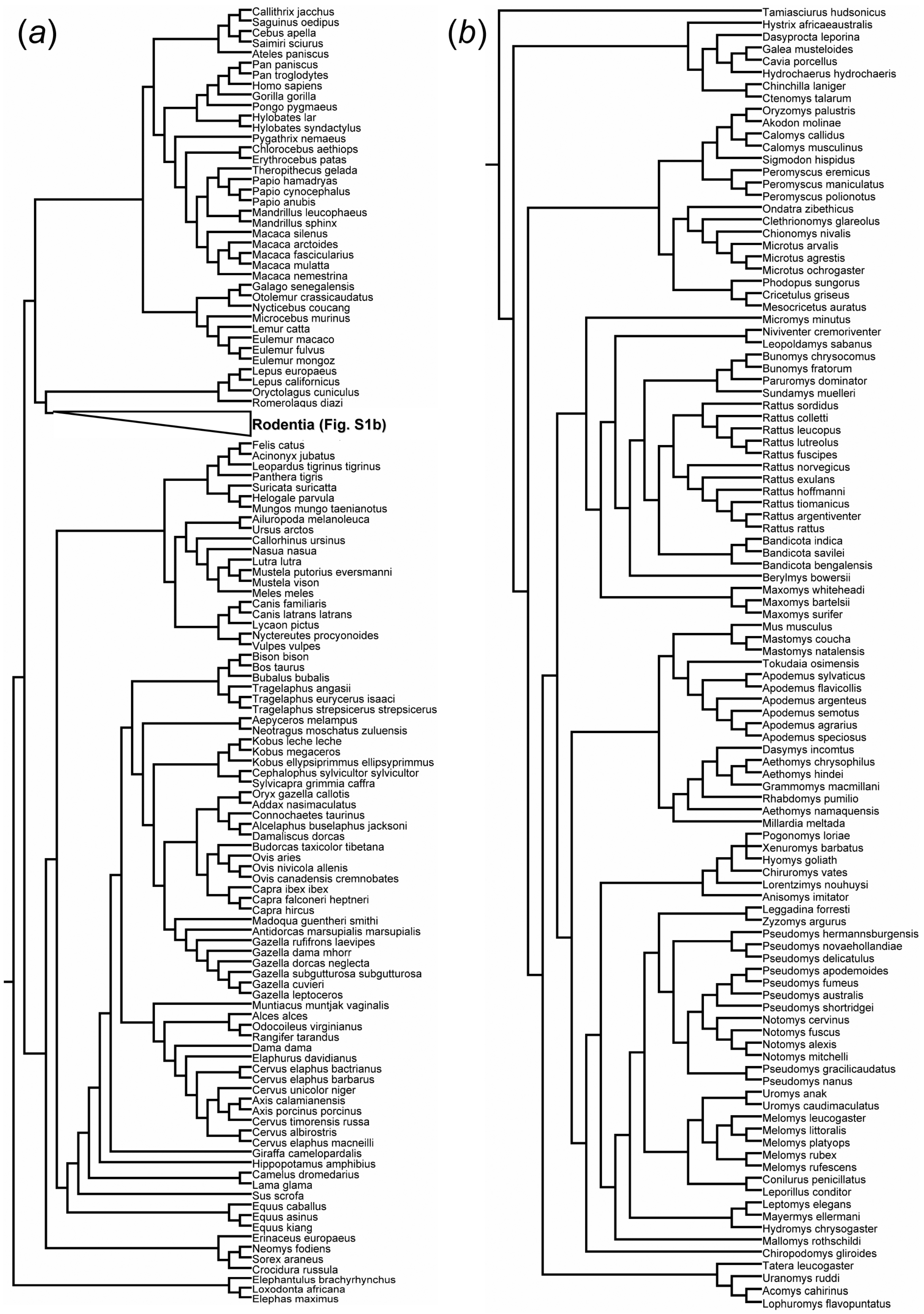
(a)
Callithrix jacchus
Saguinus oedipus
Cebus apella
Saimiri sciurus
Ateles paniscus
Pan paniscus
Pan troglodytes
Homo sapiens
Gorilla gorilla
Pongo pygmaeus
Hylobates lar
Hylobates syndactylus
Pygathrix nemaeus
Chlorocebus aethiops
Erythrocebus patas
Theropithecus gelada
Papio hamadryas
Papio cynocephalus
Papio anubis
Mandrillus leucophaeus
Mandrillus sphinx
Macaca silenus
Macaca arctoides
Macaca fascicularius
Macaca mulatta
Macaca nemestrina
Galago senegalensis
Otolemur crassicaudatus
Nycticebus coucang
Microcebus murinus
Lemur catta
Eulemur macaco
Eulemur fulvus
Eulemur mongoz
Lepus europaeus
Lepus californicus
Oryctolagus cuniculus
Romerolagus diazi
Rodentia (Fig. S1b)
Felis catus
Acinonyx jubatus
Leopardus tigrinus tigrinus
Panthera tigris
Suricata suricatta
Helogale parvula
Mungos mungo taenianotus
Ailuropoda melanoleuca
Ursus arctos
Callorhinus ursinus
Nasua nasua
Lutra lutra
Mustela putorius eversmanni
Mustela vison
Meles meles
Canis familiaris
Canis latrans latrans
Lycaon pictus
Nyctereutes procyonoides
Vulpes vulpes
Bison bison
Bos taurus
Bubalus bubalis
Tragelaphus angasii
Tragelaphus eurycerus isaaci
Tragelaphus strepsicerus strepsicerus
Aepyceros melampus
Neotragus moschatus zuluensis
Kobus leche leche
Kobus megaceros
Kobus ellypsiprimmus ellipsyprimmus
Cephalophus sylvicultor sylvicultor
Sylvicapra grimmia caffra
Oryx gazella callotis
Addax nasimaculatus
Connochaetes taurinus
Alcelaphus buselaphus jacksoni
Damaliscus dorcas
Budorcas taxicolor tibetana
Ovis aries
Ovis nivicola allenis
Ovis canadensis cremnobates
Capra ibex ibex
Capra falconeri heptneri
Capra hircus
Madoqua guentheri smithi
Antidorcas marsupialis marsupialis
Gazella rufifrons laevipes
Gazella dama mhorr
Gazella dorcas neglecta
Gazella subgutturosa subgutturosa
Gazella cuvieri
Gazella leptoceros
Muntiacus muntjak vaginalis
Alces alces
Odocoileus virginianus
Rangifer tarandus
Dama dama
Elaphurus davidianus
Cervus elaphus bactrianus
Cervus elaphus barbarus
Cervus unicolor niger
Axis calamianensis
Axis porcinus porcinus
Cervus timorensis russa
Cervus albirostris
Cervus elaphus macneilli
Giraffa camelopardalis
Hippopotamus amphibius
Camelus dromedarius
Lama glama
Sus scrofa
Equus caballus
Equus asinus
Equus kiang
Erinaceus europaeus
Neomys fodiens
Sorex araneus
Crocidura russula
Elephantulus brachyrhynchus
Loxodonta africana
Elephas maximus
(b)
Tamiasciurus hudsonicus
Hystrix africaeaustralis
Dasyprocta leporina
Galea musteloides
Cavia porcellus
Hydrochaerus hydrochaeris
Chinchilla laniger
Ctenomys talarum
Oryzomys palustris
Akodon molinae
Calomys callidus
Calomys musculinus
Sigmodon hispidus
Peromyscus eremicus
Peromyscus maniculatus
Peromyscus polionotus
Ondatra zibethicus
Clethrionomys glareolus
Chionomys nivalis
Microtus arvalis
Microtus agrestis
Microtus ochrogaster
Phodopus sungorus
Cricetulus griseus
Mesocricetus auratus
Micromys minutus
Niviventer cremoriventer
Leopoldamys sabanus
Bunomys chrysocomus
Bunomys fratorum
Paruromys dominator
Sundamys muelleri
Rattus sordidus
Rattus colletti
Rattus leucopus
Rattus lutreolus
Rattus fuscipes
Rattus norvegicus
Rattus exulans
Rattus hoffmanni
Rattus tiomanicus
Rattus argentiventer
Rattus rattus
Bandicota indica
Bandicota savilei
Bandicota bengalensis
Berylmys bowersii
Maxomys whiteheadi
Maxomys bartelsii
Maxomys surifer
Mus musculus
Mastomys coucha
Mastomys natalensis
Tokudaia osimensis
Apodemus sylvaticus
Apodemus flavicollis
Apodemus argenteus
Apodemus semotus
Apodemus agrarius
Apodemus speciosus
Dasymys incomtus
Aethomys chrysophilus
Aethomys hindei
Grammomys macmillani
Rhabdomys pumilio
Aethomys namaquensis
Millardia meltada
Pogonomys loriae
Xenuromys barbatus
Hyomys goliath
Chiruromys vates
Lorentzimys nouhuysi
Anisomys imitator
Leggadina forresti
Zyzomys argurus
Pseudomys hermannsburgensis
Pseudomys novaehollandiae
Pseudomys delicatulus
Pseudomys apodemoides
Pseudomys fumeus
Pseudomys australis
Pseudomys shortridgei
Notomys cervinus
Notomys fuscus
Notomys alexis
Notomys mitchelli
Pseudomys gracilicaudatus
Pseudomys nanus
Uromys anak
Uromys caudimaculatus
Melomys leucogaster
Melomys littoralis
Melomys platyops
Melomys rubex
Melomys rufescens
Conilurus penicillatus
Leporillus conditor
Leptomys elegans
Mayermys ellermani
Hydromys chrysogaster
Mallomys rothschildi
Chiropodomys gliroides
Tatera leucogaster
Uranomys ruddi
Acomys cahirinus
Lophuromys flavopuntatus

Phylogenetic reconstruction for the 226 eutherian mammal species utilized in the GLS analysis. This reconstruction was based on Beck et al. (2006), Nishihara et al. (2006), Bininda-Emonds et al. (2007), Kjer & Honeycutt (2007) and Springer & Murphy (2007), for the determination of phylogenetic position of the higher groups (orders and families). To resolve within groups relations the following phylogenies were used: Artiodactyla: Pitra et al. (2004), Prothero & Foss (2007), O'Leary & Gatesy (2008) and Agnarsson & May-Collado (2008); Carnivora: Veron et al. (2004), Marmi et al. (2004), Flynn et al. (2005), Johnson et al. (2006), Fulton & Strobeck (2007) and Holliday (2007); Eulipotyphla: Dubey et al. (2007); Lagomorpha: Robinson & Mathee (2005); Perisodactyla: Oakenfull et al. (2000); Primates: Steiper & Ruvolo (2003), Yoder & Yang (2004), Roos et al. (2004), Poux & Douzery (2004), Xing et al. (2005), Böhm & Mayhew (2005), Baena et al. (2007), Borges et al. (2008) and Sears et al. (2008); Rodents: Watts & Baverstock (1994), Menzies (1996), Ruedas & Kirsch (1997), Seddon & Baverstock (2000), Conroy & Cook (2000), Adkins et al. (2003), Steppan et al. (2004), Liu et al. (2004), Steppan et al. (2005), Ford (2006), Almeida et al. (2007), Swann et al. (2007), Swann (2007), Robins et al. (2007), Bradley et al. (2007), Weisbecker & Shmid (2007), Michaux et al. (2007), Morgan (2008), Rowe et al. (2008) and Lecompte et al. (2008).
